# Social determinants of cardiovascular disease outcomes in Indians

**DOI:** 10.4103/0971-5916.73415

**Published:** 2010-11

**Authors:** Panniyammakal Jeemon, K.S. Reddy

**Affiliations:** Institute of Cardiovascular & Medical Sciences, University of Glasgow, Glasgow, UK; * *Public Health Foundation of India, New Delhi, India*

**Keywords:** Acute coronary syndrome, cardiovascular disease, social determinants, social gradient

## Abstract

Cardiovascular diseases (CVD) are the leading cause of death and disability in both developed and developing countries. In developed countries socio-economic mortality differentials have been studied extensively showing that the low socio-economic group suffers the highest mortality. As the epidemiological transition is taking place against a background of economic globalization, CVD risk factors among the urban poor and middle class are rapidly increasing in India. Recent evidences from India also suggest reversal of social gradient with excess burden of CVD morbidity in the low socio-economic group. Understanding the social determinants of environmental and behavioural exposures, in determining the risk factors for cardiovascular disease is an important challenge for public health professionals as well as communities. Socio-economic disadvantage is not simply a proxy for poor cardiovascular risk factor status, but also an indication of the likely trajectory that an individual or a community may follow in the course of their life. The paucity of intervention research seeking to address the role of social determinants in shaping lifestyle practices among individuals in culturally and socially diverse population groups within India is definitely a measure of inadequacy in public health research. This review article provides an overview of the role of social determinants of CVD and its possible conceptual pathways with special focus on acute coronary syndrome (ACS) outcomes among Indians.

## Introduction

Cardiovascular disease (CVD) is no longer limited to developed countries, it is widespread in developing countries^1^,^2^. Additionally, the rate of increase in CVD in developing countries is twice as high as in developed countries^3^. CVD also typically occurs at a younger age in developing countries: for example, about 52 per cent of deaths from such disorders in India occur before 70 yr of age, compared with 23 per cent in established-market economies^4^. In the context of this large and growing disease burden, strategies to improve population health in India require consistent efforts to identify and address the real causes of this rapid rise in CVD.

In developed countries socio-economic mortality differentials have been studied extensively showing that the low socio-economic group suffer the highest mortality^5^–^10^. Such a trend was not observed for CVD in India until the 1990s, and CVD was regarded as a disease of the affluent classes^11^. The epidemic of CVD struck the more affluent sections of India first, but as the epidemic is maturing, we are observing a graded reversal of social gradient, with socio-economically disadvantaged groups becoming increasingly vulnerable to CVD. For example, the social class gradient in cardiovascular event rates among Indians has reversed with evidence for excess CVD events among the lower socio-economic groups^12^–^14^. In a survey conducted in 45 rural villages in India, 32 per cent of all deaths were due to CVD, outranking infectious diseases, which were responsible for 13 per cent giving clear evidence that the epidemic has reached its advanced stage even in rural India^15^. Neglect of this epidemic, particularly ignoring the socio-economic context as they are driven by social and economic changes with lack of an adequate public health response will further fuel the inequities associated with CVD in Indians.

Socio-economic mortality differentials have been demonstrated using several indicators of social position representing occupational, educational, and financial aspects^16^. Commonly used variables for assessing social determinants of health are given in Table I. Although most of the studies have considered each socio-economic variable individually and at single points in time, the mechanisms through which these affect health are often linked and vary over time. It is challenging to quantify the effect of social disadvantage on CVD outcomes because social and economic factors are surrogates for the latent construct of social disadvantage. While the various dimensions of socio-economic position are interrelated, it has been suggested that these represent rather different forces associated with health outcomes^16^. However, education has been proposed as the preferable variable to other socio-economic indicators to describe health outcomes. There is evidence from several countries showing that its association with mortality and morbidity is probably the strongest among other social determinants of health^17^–^19^. This paper provides an overview of the role of social determinants of CVD and its possible conceptual pathways with special focus on acute coronary syndrome (ACS) outcomes among Indians.

**Table I:** Variables for the measurement of social determinants of health

Indicator	Variables
Place of residence	Location of residence or neighbourhood of residence (rural, urban, peri-urban), citizenship, house ownership, *etc*.
Race/Ethnicity	Race, ethnicity, dissimilarity index*, isolation index**, perceived discrimination, foreign born status, *etc*.
Gender	Gender, partner violence, perceived role within family, perceived discrimination against males/females, sex selective abortion rates, *etc*.
Education	Highest educational attainment, years of education, literacy rate, *etc*.
Socio-economic status	Income, education, occupation, parent’s education, parent’s occupation, household items, type of house, ownership of land, car and other luxury items, *etc*.

* The relative separation or integration of groups.

** The probability that a member of one group will meet another member of the same group

## Social class differences in CVD outcomes: evidence from migrant studies

Among South Asians in the United Kingdom (UK), there is a continuing excess of CVD and in particular ischaemic heart disease (IHD) deaths^20^–^21^. A literature review of all published studies also suggest significantly higher coronary heart disease (CHD) mortality ratio of 1.1-3.8 among migrants from Indian subcontinent when compared to the host country populations^22^. Two decades ago, McKeigue *et al*^23^ linked this excess CHD risk in migrant South Asians in UK to an emerging trend in inverse association between social class and CHD^23^. Later, Bhopal *et al*^24^ demonstrated this relationship in migrants from the Indian sub-continent in UK. In a recent study, Tillin *et al*^25^ present evidence on increased risk of ischaemic heart disease (IHD) when socio-economic disadvantage was measured as fewer years of education among predominantly Indian, South Asian community in UK^25^. While Tillin’s study highlights the intimate relationship between CVD outcomes and social class, similar observations on social class differences in perceived health outcomes among Indians in UK have been reported by Chandola^26^. This clearly indicates that social class differences are emerging as important determinants of increased CHD risk in migrants from the Indian sub-continent.

## Social determinants of CVD outcomes in India

Case control studies of acute myocardial infarction (AMI) in India showed significantly higher risk of AMI among low socio-economic group compared to high socio-economic group^12^,^13^. Similarly, 30 days follow-up data of acute coronary syndrome (ACS) patients from CREATE registry demonstrated significantly higher mortality rate in low socio-economic status (SES) group compared to higher SES group^14^. Further, a clear inverse gradient of SES and ACS related mortality was evident across four different categories of socio-economic position. These differences persist even after adjustment for clinical symptoms and the area of infarct. The SES inequity in risk for ACS mortality is not fully accounted for by socio-economic gradients of classical risk factors in CREATE registry, which suggests additional or alternative pathways underlie the association between SES and cardiovascular diseases. However, the inverse gradient disappeared once it was adjusted for treatment differences across various socio-economic groups. Three quarters of patients in this study were from lower middle socio-economic class and poor backgrounds. They were either less likely to afford the treatments in the hospitals for secondary prevention or the health care personnel were inept in identifying the needs of this group due to various other reasons.

## Gradient in CVD risk factors across socio-economic groups

As the epidemiological transition is taking place against a background of economic globalization, CVD risk factors among the urban poor and middle class are rapidly increasing in India. Consequent to the impact of globalization, there is an “aspiration effect” which has behavioural consequences among the upper middle class and rich people in India. The aspiration for to emulate the western society leads to improved awareness of the preventive measures to tackle the raised CVD risk in these groups. By contrast, higher levels of tobacco use, obesity or overweight and hypertension are now associated with lower levels of education and income in India^27^,^28^. Since development is socially and regionally uneven in India, the social gradient in CVD risk factors is also expected to be uneven across societies and geographical locations. For example, in more urbanized communities there is higher vulnerability of low educational group to CHD risk factors and related events compared to less urbanized communities in India^29^. It is therefore, important to understand that the socio-economic disadvantage is not simply a proxy for poor cardiovascular risk factor status, but also an indication of the likely trajectory that an individual may follow in the due course of life.

## Social gradient in awareness and treatment seeking behaviours related to CVD risk factors

While prevalence of diabetes in India increases as the educational level decreases, the awareness of diabetes was lowest in the low education group^30^. Several urban-rural comparison studies also documented poor awareness of CVD risk factors in socio-economically disadvantaged rural population^31^–^36^. These studies also highlight the inadequate treatment practices for management of risk factors especially diabetes and hypertension among the rural population. Widespread implementation of programmes like hypertension detection and follow up programme in US resulted in increased awareness, treatment and control of hypertension and ultimately eliminated the association of mortality with low socio-economic status^37^. Similar community based programmes will be helpful for reducing the cardiovascular disease burden in Indian population as well.

## Conceptual models for social gradient in health outcomes

*The latent effect model*: The latency model emphasizes the prospect that psychosocial and socio-economic conditions vary early in life will have a strong impact later in life independent of intervening experience. Power & Hertzman^38^ describe a latent effects model wherein certain early life events may have strong independent effects on adult health. However, circumstances in early childhood can affect health status later in life independent of intervening experience (*i.e*., latent effects), and also through the life pathways that individuals get set on (*i.e*., pathway effects).

*The pathway model*: The pathway model emphasizes the cumulative effect of life events and the reinforcing effect of differing psychosocial and socio-economic circumstances throughout the life cycle. The duration of “exposure” to at-risk living conditions has a dose-response effect on subsequent health and well-being. Hertzman *et al*^39^ propose a developmental process linking early-life psychosocial environments with adult health risk via pathway effects, wherein early experiences place an individual onto a certain “life trajectory,” eventually impacting adult health. While a pathway life course model is generally appealing, its operation is difficult to test empirically. Life course studies typically collect information on participants at two or three time points, which do not permit the continuous, lifelong operation of pathway effects to be observed.

*The social mobility model*: Forsdahl hypothesized that deprivation in early life followed by later affluence combine to produce elevated CVD mortality risk, partly via elevation of adult cholesterol levels^40^. According to this hypothesis, migrants who encounter prosperity in adult life, having experienced relative deprivation during childhood, should face an even greater risk of CVD than long-term residents of the country they migrate to. However, early deprivation may not be the only explanation for high CVD rates in South Asian migrants in UK, since Afro-Caribbean’s and others who have migrated from less developed regions have low CVD mortality rates^20^.

*The cumulative model*: The cumulative SES life course model hypothesizes that psychosocial and physiological experiences and environments during early and later life accumulate to influence adult disease risk. Smith *et al*^41^ suggest that if factors operating at different life stages are combined, large differences in CVD risk will be observed. An additive effect of both childhood and adulthood socio-economic disadvantage on CVD mortality has been reported in the Southall study^25^.

## New perspectives on social gradients in health outcomes

The social gradient in health (health status rises with each level of socio-economic status) suggests that health status may be embedded in collective factors in society, not just in individual factors. Since health status follows a gradient pattern, people in poorer socio-economic circumstances are not as healthy as those in the middle-class, and middle-class groups are not as healthy as those at the top. However, a large number at the bottom still develop into healthy and competent adults. In other words, not all children and adults living in low socio-economic circumstances have poor health, they are simply more likely to develop poor physical and emotional outcomes than those living in better circumstances.

The socio-economic gradient in health status is not new in India. In fact, the socio-economic gradient in health status seems to be able to replicate itself on the principal diseases of each era, despite the fact that their pathological basis varies greatly. For instance, till early 1990s the gradient was found for infectious diseases that were the principal cause of death in India at that time. By the beginning of the new century, the socio-economic gradient had replicated itself in heart diseases which are the current major causes of death^3^,^4^,^15^.

## Future research needs

Possible study designs to understand the social gradient in CVD morbidity and mortality are given in the Table II. Longer prospective studies or series of cross-sectional studies that evaluate associations between early-life experiences and risk factor levels at several time points may provide the opportunity to observe the operation of pathway life course effects. Life course SES studies have generally failed to consider the length of exposure to the various socio-economic conditions measured. As this may influence the impact of negative SES experiences on adult health, future cumulative life course studies may benefit from evaluating the effect of length of exposure into their indices. The paucity of intervention research seeking to address the role of social determinants in shaping lifestyle practices among individuals in culturally and socially diverse population groups within India is definitely a measure of inadequacy in public health research. A large opportunity for pioneering evaluation research models that will demonstrate the effectiveness of social determinant interventions in reducing and ultimately eliminating health disparities is therefore possible in countries like India.

**Table II:** Study designs on social determinants in CVD morbidity and mortality

Study hypothesis	SES variable	Outcome variable	Study design/s
CVD event rates are different in diverse SES groups	Present SES	CVD event rates	Ecological analysis
			Disease registries
			Cohort study
CVD risk factors are different in diverse SES groups	Present SES	Prevalence/incidence of CVD risk factors	Ecological analysis
			Cross-sectional surveys
			Serial epidemiological surveys
			Cohort study
Adult risk of CVD is different in SES groups based on early life socio-economic position	Early life SES (childhood SES, paternal/maternal SES	CVD risk factor prevalence/incidence	Serial epidemiological surveys
			Cohort study
CVD outcomes are different in rural to urban or country to country migrants and non-migrants based on their socioeconomic position before and after migration	SES and inter- intrageneration movement	CVD risk factor prevalence/incidence	Cohort study
			Multi-level analysis*
CVD outcomes are different according to the number of negative exposures in the life course	Negative SES exposures at different time points	CVD risk factors	Cohort study
		CVD events	Multi-level analysis*

* Multilevel analyses integrate individual-level variables with group- and macro-level variables so that multiple levels of influence can be assessed simultaneously

## Policy implications of socio-economic gradient in CVD outcomes in Indians

Socio-economic determinants are strongly linked to CVD risk factors, related morbidity and mortality. Failure to acknowledge, and more importantly, to understand the role of social determinants in health and access to health and social services will hamper any effort to improve the health of the population. A paradigm shift away from the biomedical model is therefore required in the perspective of the existing health care system while responding to the rapidly increasing burden of CVD morbidity and mortality in India. A population perspective, that considers social and ethnic differences in health status, needs to be highlighted and the action plan should involve institutions outside the health sector, based on an appreciation of the economic and social causes of disease. Persistence and an open attitude to learning without receding to previous paradigms and ways of intervening, can lead us to uncover contemporary ways to address the issues that underlie and perpetuate socio-economic disparities in CVD outcomes. Identifying and addressing disparities based on socio-economic position will surely move India closer to the Millennium Development Goals (MDGs).
